# Cardiac CT Beyond Coronaries: Focus on Structural Heart Disease

**DOI:** 10.1007/s11897-023-00635-9

**Published:** 2023-11-29

**Authors:** Michaela M. Hell, Tilman Emrich, Philipp Lurz, Ralph Stephan von Bardeleben, Axel Schmermund

**Affiliations:** 1 Department of Cardiology, University Medical Center Mainz, Johannes Gutenberg University, Mainz, Germany; 2 Department of Diagnostic and Interventional Radiology, University Medical Center Mainz, Johannes Gutenberg-University, Mainz, Germany; 3 Cardioangiologisches Centrum Bethanien, Frankfurt am Main, Germany

**Keywords:** Computed tomography, Structural heart disease, Valvular heart disease, Transcatheter valvular interventions

## Abstract

**Purpose of Review:**

Cardiac computed tomography (CT) is an established non-invasive imaging tool for the assessment of coronary artery disease. Furthermore, it plays a key role in the preinterventional work-up of patients presenting with structural heart disease.

**Recent Findings:**

CT is the gold standard for preprocedural annular assessment, device sizing, risk determination of annular injury, coronary occlusion or left ventricular outflow tract obstruction, calcification visualization and quantification of the target structure, and prediction of a co-planar fluoroscopic angulation for transcatheter interventions in patients with structural heart disease. It is further a key imaging modality in postprocedural assessment for prosthesis thrombosis, degeneration, or endocarditis.

**Summary:**

CT plays an integral part in the imaging work-up of novel transcatheter therapies for structural heart disease and postprocedural assessment for prosthesis thrombosis or endocarditis. This review provides a comprehensive overview of the key role of CT in the context of structural heart interventions.

## Introduction

Coronary computed tomography (CT) angiography has been established as an important application of cardiac CT imaging, because the method is well suited for visualizing the small coronary vessels that are always in motion [1••]. Its high temporal and spatial resolution, inherently three-dimensional character, and ready availability have rendered it increasingly attractive also for non-coronary cardiac imaging [2••]. The latest milestone in CT technology is the photon-counting detector system which is capable of an even increased spatial resolution, absence of electronic noise, and multienergy capability [3]. The vast majority of non-coronary CT studies pertain to the aortic valve, mainly in preparation for transcatheter aortic valve implantation (TAVI) or for prosthetic aortic valve follow-up examinations [4••]. Other applications involve characterization of mitral or tricuspid valve, left atrial appendage (LAA) anatomy with surrounding structures, paravalvular leaks (PVL) after surgical or interventional prosthetic valve replacement, or the identification of the optimal transseptal puncture site [5, 6, 7•, 8, 9]. In addition, CT is recommended for endocarditis diagnostics in prosthetic valves [10••, 11, 12]. Advanced applications for CT in structural heart disease as fusion imaging or computational fluid dynamics for providing cardiac blood flow patterns have been described in detail elsewhere [13]. Though this review focuses on CT, it is important to mention that imaging in the context of structural heart disease involves a multimodality approach with echocardiography as the primary imaging modality to identify and grade the severity of a valvular lesion [14]. The particular strength of CT here is the isotropic resolution allowing to reconstruct data sets in any desired plane without losing the ability to perform exact measurements and the visualization of calcifications. The current review will focus on these above-mentioned applications, because just as coronary CT angiography uniquely fits into the important niche of non-invasive coronary imaging, they represent a unique usage of non-coronary CT, a “structural heart disease niche.”

## General Aspects on CT Image Acquisition for Structural Heart Disease

CT scanning protocols for imaging the aortic, mitral, and tricuspid valve as well as the LAA have been discussed in detail in current literature [4••, 15, 16]. The exact specifications of the CT protocols differ according to the planned heart valve procedure and the scanner used, but some general principles should be taken into consideration: It is recommended to use at least a 64-slice CT scanner and a slice thickness of 0.6–0.75 mm. In order to perform accurate measurements of the cardiac target structure, an ECG-synchronized, contrast-enhanced CT data set is recorded over the entire cardiac cycle with only moderate tube current modulation to maintain image quality throughout diastole. Non-ECG-synchronized CT angiography is sufficient to assess the access route including the thoracic and abdominal aorta, iliac and common femoral vessels, and, if necessary, also subclavian and jugular vessels. Due to frequent renal comorbidities and the higher age of these patients, use of iodinated contrast media should be applied with caution, generally given at a rate of 4–6 ml/s, amounting to a total of 50–100 ml. Heart rate control using beta blockers is not generally recommended in patients with severe aortic stenosis due to the risk of potential side effects. In patients with severe mitral or tricuspid regurgitation, low-dose negative chronotropic/inotropic agent is generally tolerated and should be considered if heart rates are above 100 beats/min. Nitroglycerin is contraindicated in patients with severe aortic stenosis.

## General Aspects on the Systematic Evaluation of the Target Structure

When analyzing the acquired CT data sets, it is of particular importance that measurements are performed in an imaging plane that is exactly aligned with the target structure. Multiplanar reconstructions with interactive manual manipulation of imaging planes require sufficient experience and are reasonable for aortic valve assessment [17]. Conversely, the complex three-dimensional (3D) non-planar anatomy of the atrioventricular valves requires mostly 3D measurements using specific software solution products for image processing and procedural planning [18]. CT-derived 3D computational modeling for virtual implantation of catheter-based devices and 3D printing models for device evaluation in life-size replicas of patient-specific cardiac anatomy have been introduced [19].

## CT-Based Procedural Planning of Transcatheter Valvular Interventions

### CT for Planning Transcatheter Aortic Valve Implantation

Worldwide, since the advent of TAVI in 2002, >1.5 million procedures have been performed [20]. Whereas initially only patients deemed to be at high surgical risk were treated by using TAVI, recent studies with excellent outcomes also in patients with intermediate and low surgical risk have led to expanding indications. Current studies focus on patient age (> 65 years), life expectancy, comorbidity, and suitable anatomy [21].

Comprehensive recommendations regarding CT imaging in the context of TAVI have been published by the Society of Cardiovascular Computed Tomography [4••]. The key aspects of CT imaging before TAVI are listed in Table 1.

**Table 1:** Key aspects of CT imaging for planning transcatheter valve interventions

Transcatheter aortic valve implantation	• Assessment of aortic valve anatomy and morphology (e.g., bicuspid valve) • Assessment and sizing of the aortic root • Visualization and quantification of annular and LVOT calcifications • Evaluation for risk of coronary artery obstruction • Prediction of optimal fluoroscopic projection angles for device implantation • Assessment of vascular access route • Additional: virtual device simulation
Transcatheter mitral valve interventions	• Assessment of mitral valve anatomy and morphology (height and angle of the tenting of the mitral leaflets) • Assessment and sizing of mitral annulus • Visualization and quantification of mitral annular calcification • Prediction of Neo-LVOT and evaluation for risk of LVOT obstruction • Assessment of the device landing zone • Sizing of the left ventricular heart cavities • Determination of access route (transseptal puncture location or transapical route) • Prediction of optimal fluoroscopic projection angles for device implantation • Virtual device simulation
Transcatheter tricuspid valve interventions	• Assessment of tricuspid valve anatomy and morphology • Assessment of anatomic relationships with surrounding structures and possible impediments (e.g., right coronary artery) • Assessment of the device landing zone • Assessment of dimensions and function of the right ventricle (RV) • Prediction of an optimal fluoroscopic projections for device implantation • Assessment of the vascular access route • Virtual device simulation • Heterotopic caval valve implantation: assessment of dimensions of the inferior vena cava, distance between the junction plane of the inferior vena cava and right atrium and first hepatic vein, size of right atrium
Transcatheter left atrial appendage (LAA) occlusion	• Assessment of LAA anatomy and morphology (e.g., accessory lobes, shape) • Measurement of LAA dimensions • Assessment of the device landing zone • Assessment of anatomic relationships with surrounding structures • Assessment of the interatrial septum and prediction of an optimal fluoroscopic projections for transseptal puncture • Assessment for thrombus

The aortic annulus is defined by the basal attachment points of the aortic cusps. These so-called basal hinge points need to be accurately defined by CT imaging, and there are manual or software-based algorithms available for this purpose [4••]. The contours should be drawn, either manually or by using software-based algorithms, along the interface between the blood pool and tissue, yielding the area and perimeter. An eccentricity index can be derived from long and short-axis dimensions. Of note, the largest annular area throughout the cardiac cycle should be determined, mostly corresponding to frames during systole. In the presence of severe septal hypertrophy, systolic bulging of the interventricular septum may lead to a paradoxical decrease in annular size in systole [4••]. Undersizing of the prosthetic valve may result in aortic regurgitation, underlining the importance of correct measurements of the annulus. Equally important, severe annular and LVOT calcification needs to be characterized, as it may also lead to prosthesis malfunction or, even worse, ostial occlusion of the coronary arteries during implantation of the valve. Finally, the delineation of valve morphology is necessary in particular with regard to a bicuspid anatomy, which is found in up to 6% of patients undergoing TAVI [4••].

In patients with a low coronary ostial height, the prosthesis can potentially occlude the ostia. Even in the early experience, this has been described in <0.5–1% of patients, but mortality is high [22]. Accordingly, procedure planning must take into account coronary ostial height for choosing the optimal prosthesis. In general, a low coronary ostial height < 12 mm and a sinus of Valsalva diameter < 30 mm is accompanied with an increased risk of coronary ostial occlusion [4••].

With the assumption that patient positioning is similar in the CT scanner and intraprocedurally on the catheterization laboratory table, optimal fluoroscopic angulations can be derived from the CT images, showing the aortic annulus either in the classical co-planar view or the alternative cusp-overlap view sometimes preferred for self-expandable valves [4••, 23].

There has been a constant reduction of sheath size, allowing transcatheter femoral access in the vast majority of patients. Increased operator’s experience in the management of the vascular access as well as technological refinements of current device generations has reduced the rate of vascular complications to <10% [24]. CT imaging has been established as a prerequisite for avoiding access site complications and, if necessary, to choose an alternative access route [4••]. Vascular dimensions can be described and potential challenges be detected such as stenosis or occlusion, calcification, aneurysm or dissection, and tortuosity. Intraprocedurally, ultrasound guidance is generally used for controlling the puncture zone [25].

With the recent extension of TAVI indications to lower surgical risk patients with increased life expectancy, concerns about the prosthesis durability and the postprocedural coronary access have become even more important issues. Commissural alignment, as routinely performed in surgical aortic valve replacement, has been identified as an important step for TAVI procedure optimization in terms of coronary access, valve hemodynamics, coronary flow, and also access after redo-TAVI [26, 27]. Preprocedural planning with CT for commissural alignment includes the identification of patient-specific fluoroscopic projections as well as important anatomic information on the coronary cusps, native commissures, and coronary ostia. Post-TAVI CT is the gold standard for evaluation of commissural alignment by measuring the angle between the THV commissural post and native aortic valve commissure. The ALIGN-TAVR (Alignment of Transcatheter Aortic-Valve Neo-Commissures) consortium has defined commissural alignment according to the commissural offset using a four-tier scale [26]. As this examination requires additional contrast injection and radiation, it is a not routinely performed.

### CT for Planning Transcatheter Atrioventricular Valve Interventions

In contrast to the tubular, planar structure of the aortic valve apparatus, the mitral and tricuspid valves show a complex saddle-shaped anatomy of leaflets, annulus, and subvalvular apparatus. They undergo significant changes in size and geometry during the cardiac cycle. This makes the development and planning of interventional treatment methods for these defects more challenging than for the aortic valve. A thorough understanding of the atrioventricular valve anatomy and geometry is therefore essential. CT is routinely performed for transcatheter direct annuloplasty planning and valve replacement. For transcatheter edge-to-edge repair therapy, CT use is based on individual consideration.

### CT for Planning Transcatheter Mitral Valve Procedures

**Fig. 1:**
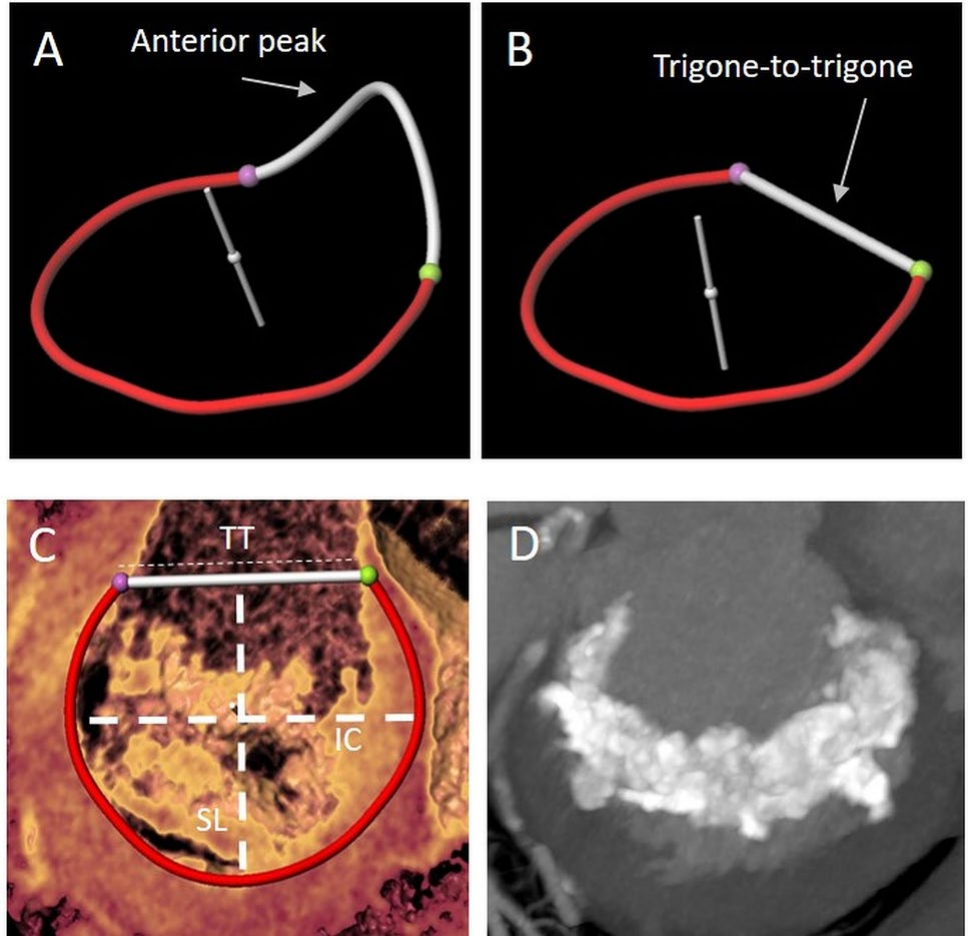
CT for planning transcatheter mitral valve procedures. **A**–**C** Mitral annular segmentation (pink dot = medial fibrous trigone; green dot = lateral fibrous trigone) using a semiautomated software approach. **A** Saddle-shaped mitral annulus with the anterior horn. **B** The “D-shape” mitral annulus is created by truncating the anterior horn at the trigones resulting in a more planar geometry. **C** Determination of annular measurements for preprocedural planning (IC, intercommissural distance; SL, septal-lateral distance; TT, trigone-trigone distance). **D** Short-axis maximum intensity projection showing severe mitral annular calcification

CT is an essential imaging modality for patient selection and procedural planning of transcatheter interventions in the non-calcified and calcified native mitral annulus as well as in prosthetic valves and rings [28]. Precise procedural planning includes annulus sizing, an anatomical and geometrical assessment of the device landing zone, its relationship to important adjacent structures, the prediction of the Neo-left ventricular outflow tract (LVOT), and an assessment of mitral annular calcification (Table 1, Fig. 1) [5]. Furthermore, CT provides information on the height and angle of the tenting of the mitral leaflets as well as the dimension and size of the left heart cavities.

The saddle-shaped, non-planar structure of the mitral annulus makes it difficult to establish a two-dimensional CT model for procedure planning (Fig. 1A). As a simplification, the “D-shape” configuration was introduced, in which the two trigone fibrosa are connected by a virtual line and form a “D” with the posterior portion of the mitral annulus, neglecting the anterior horn (Fig. 1B, C) [29]. This configuration corresponds better to the planar landing zone of the prosthesis and reduces the risk of prosthesis oversizing and LVOT obstruction (comparatively smaller projected area). Though manual segmentation can be performed to identify the two fibrous trigones, 3D measurements requiring semiautomated software are highly reproducible and used for procedural planning in most cases [30].

A central task of CT planning for transcatheter mitral valve replacement is the prediction of the Neo-LVOT and the risk assessment of a potential LVOT obstruction by virtual device simulation [31]. The implantation of the prosthesis results in a change of the LVOT geometry, which is formed by the displaced anterior mitral leaflet, the prosthetic stent, and the basal-mid anteroseptal wall of the left ventricle. A feared complication is its relocation and narrowing resulting in a relevant LVOT obstruction. Predictors for a LVOT obstruction are, in particular, an increased protrusion of the prosthesis into the left ventricle, a narrow aortomitral angle, a small cavity size of the left ventricle, and a pronounced basal septal hypertrophy [32].

Mitral annular calcification (MAC) is a degenerative process affecting the mitral valve support structure and is reported in up to 15% of patients based on postmortem investigations [33]. It is a challenging condition for surgical treatment due to frequent comorbidities and an increased risk of atrioventricular groove rupture, circumflex artery injury, or embolism. CT has been established as the gold standard for MAC detection and quantification (Fig. 1D) [34]. The CT-based MAC score by Guerrero *et al.* is a dedicated approach to evaluate MAC severity for Valve-in-MAC procedures using balloon-expandable protheses. It takes into account calcium thickness, calcium distribution in the annulus circumference, and calcification involving the trigones and leaflets [35]. With a maximum of 10 points, a score of ≤3 is classified as mild MAC, 4 to 6 as moderate MAC, and ≥7 as severe MAC. Prosthesis embolization or migration was lower with increasing MAC score, identifying a score of ≤6 points as an independent predictor of prosthesis embolization or migration in transcatheter Valve-in-MAC procedures.

### CT for Planning Transcatheter Tricuspid Valve Procedures

Modern devices and a better hemodynamic understanding of the right heart have revolutionized the treatment options for patients with severe tricuspid regurgitation in recent years. The therapeutic approaches and available devices for transcatheter tricuspid valve interventions include edge-to-edge repair, direct annuloplasty, orthotopic and heterotopic valve replacement [36, 37].

CT-based procedure planning enables the determination of the annulus size, height, and angle of tenting of the tricuspid valve leaflets, assessment of the device landing zone, the right ventricular heart cavity dimensions, and the relationship to important neighboring structures (Table 1). Valve calcification, on the other hand, is very rare in the low-pressure system. When acquiring CT data, optimal contrast enhancement of the right heart must be ensured. Several approaches to provide excellent opacification of the right heart have been published [15, 38].

**Fig. 2:**
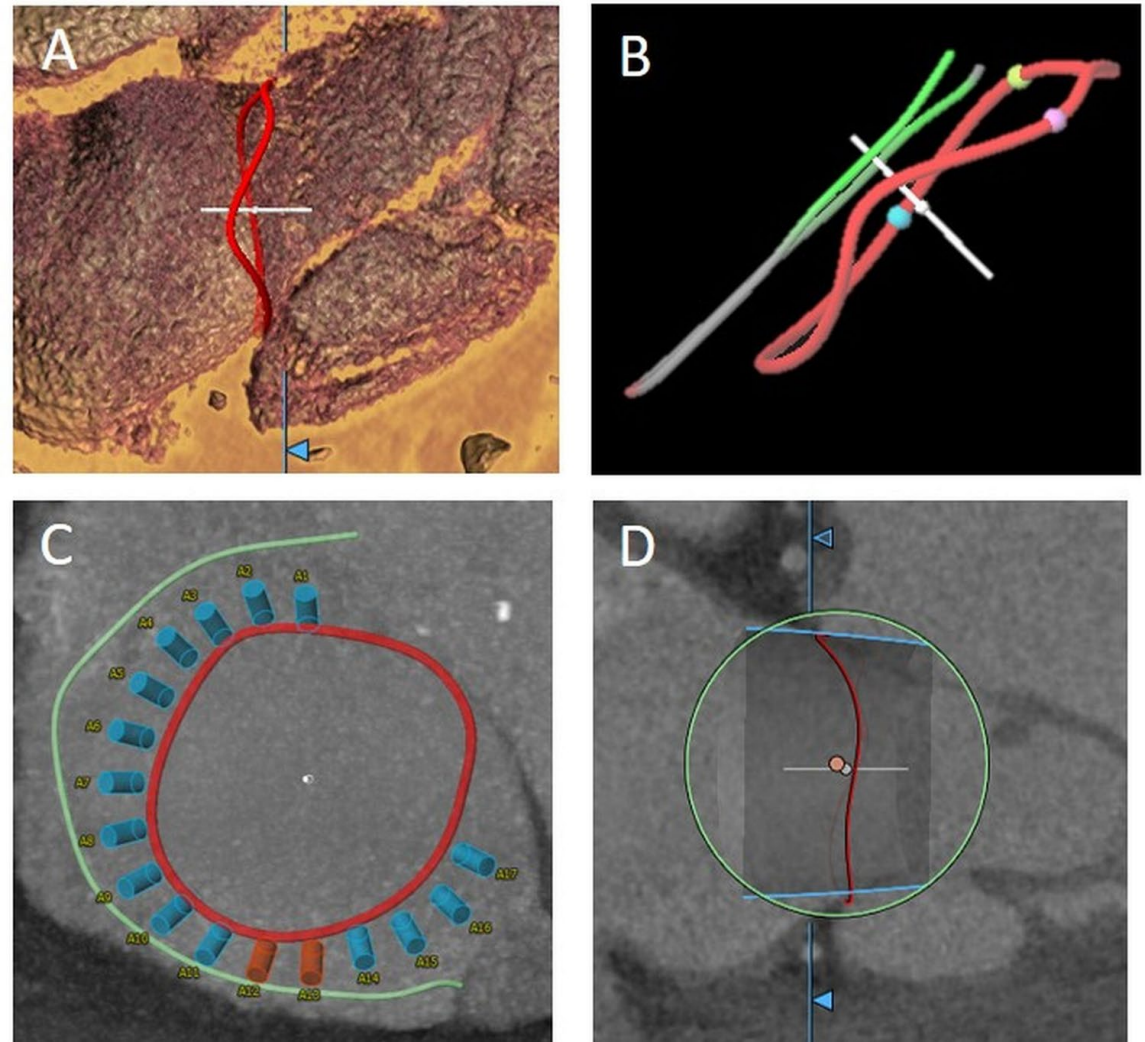
CT for planning transcatheter tricuspid valve procedures. Tricuspid annular segmentation. **A** 3D annular view. **B** CT-derived fluoroscopic view of the tricuspid annulus (red line) and the right coronary artery (RCA, green/grey line). The RCA has a superior (atrial) course in relation to the annulus. **C** CT assessment for transcatheter direct annuloplasty repair. View on the tricuspid annulus (red) with simulation of anchored annuloplasty device (blue and orange anchors) in relation to the RCA course (green). While blue anchors have a distance of ≥4 mm to the RCA, orange anchors with a distance of <4 mm have a higher risk for RCA impairment. **D** Simulation of orthotopic transcatheter tricuspid valve replacement

A 3D-based approach using semiautomated software is recommended for annulus sizing, since 2D measurements lead to an underestimation due to the complex non-planar annulus geometry (Fig. 2A) [39]. Because of the dynamic changes during the cardiac cycle, measurements should be taken in both end-systole and mid-diastole [40].

The right coronary artery is closely anatomically related to the tricuspid annulus and can be injured during transcatheter annuloplasty. The course of the right coronary artery and the distance to the annulus is therefore routinely evaluated by CT (Fig. 2B). In addition, a virtual simulation of the implantation may be performed for predicting optimal placement of the device (Fig. 2C, D).

### CT for Evaluation of Valve Prosthesis

**Fig. 3:**
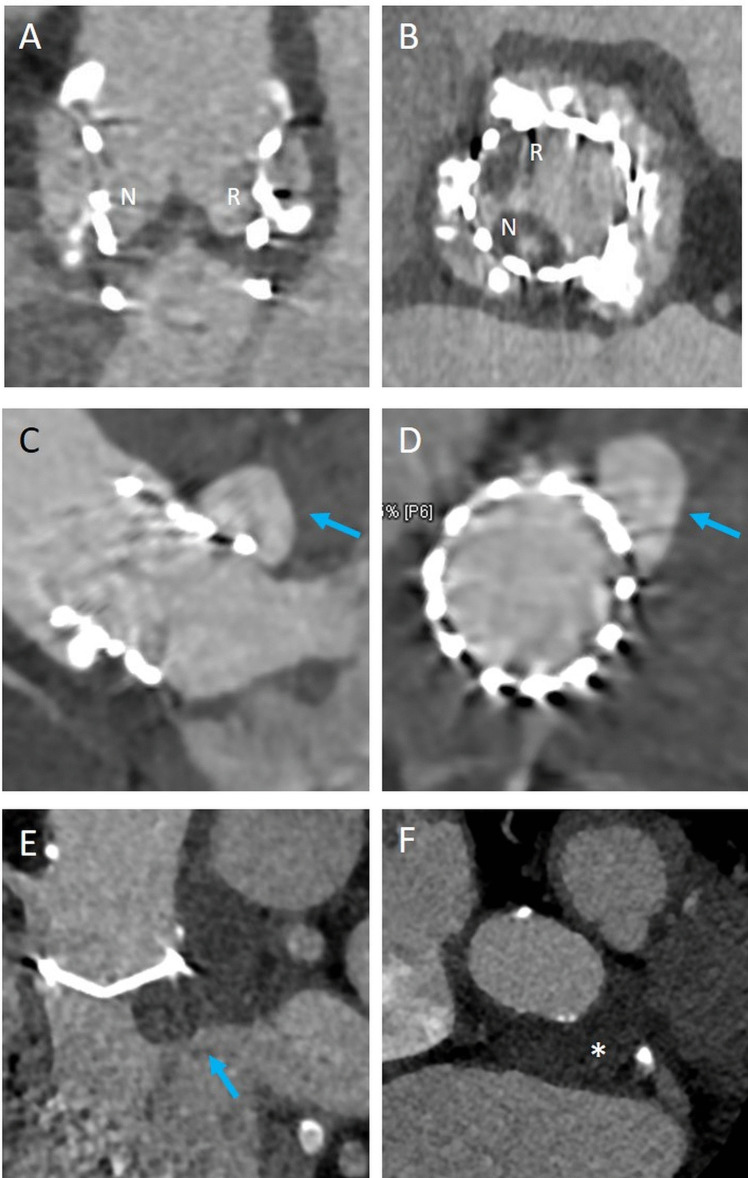
CT for evaluation of valve prosthesis. **A**, **B** Hypoattenuated leaflet thickening (HALT) affecting the Neo-NCC (N) and Neo-RCC-leaflet (R) of the TAVI prosthesis. **C**, **D** Masked aortic rupture with pericardial effusion (blue arrow; *Courtesy Philipp Breitbart, MD, University Heart Center Bad Krozingen).* **E**, **F** Patient with mechanical aortic valve and large vegetation (blue arrow) on the anterior leaflet with a large paravalvular abscess (asterisk)

Performing a CT after interventional heart valve therapy is not routine diagnostics, but should be considered if there is an echocardiographic suspicion of leaflet thrombosis, endocarditis, degeneration of the prosthesis, or masked aortic rupture with pericardial effusion (Fig. 3).

Although leaflet thrombosis has also been observed after surgical bioprosthetic aortic valve replacement, TAVI in particular may be associated with subclinical leaflet thrombosis, with or without restricted leaflet motion [41]. The reference method for detecting hypoattenuated leaflet thickening (HALT) and/or hypoattenuation affecting motion (HAM) is CT [42, 43] (Fig. 3A, B). Surprisingly, current data do not appear to indicate an association of HALT or HAM with untoward clinical events, even though at least temporary hemodynamic deterioration of valve function can be observed [44]. In asymptomatic patients with normal echocardiographic valvular function, CT follow-up examinations are therefore currently not mandated. However, in case of echocardiographic abnormalities, CT may be very useful.

Contemporary assessment of infective endocarditis contains an integrated multimodality approach with a central role of echocardiography and additional CT, 18-FDG positron emission tomography, or cardiac magnetic resonance imaging, individually guided by the Heart Team [10••, 11, 12]. CT shows a high accuracy for large vegetations (≥10 mm) and is superior to echocardiography for detection of perivalvular complications (anatomy of pseudoaneurysms, abscesses, or fistulae) (Fig. 3E, F).

## Further Applications of CT for Structural Heart Disease

Site-specific transseptal puncture is required in many left-sided heart interventions. In complex anatomies, CT allows additional insights on interatrial septum characteristics, assessment of adjacent structures, and prediction of fluoroscopic angulation in addition to echocardiography [8].

**Fig. 4:**
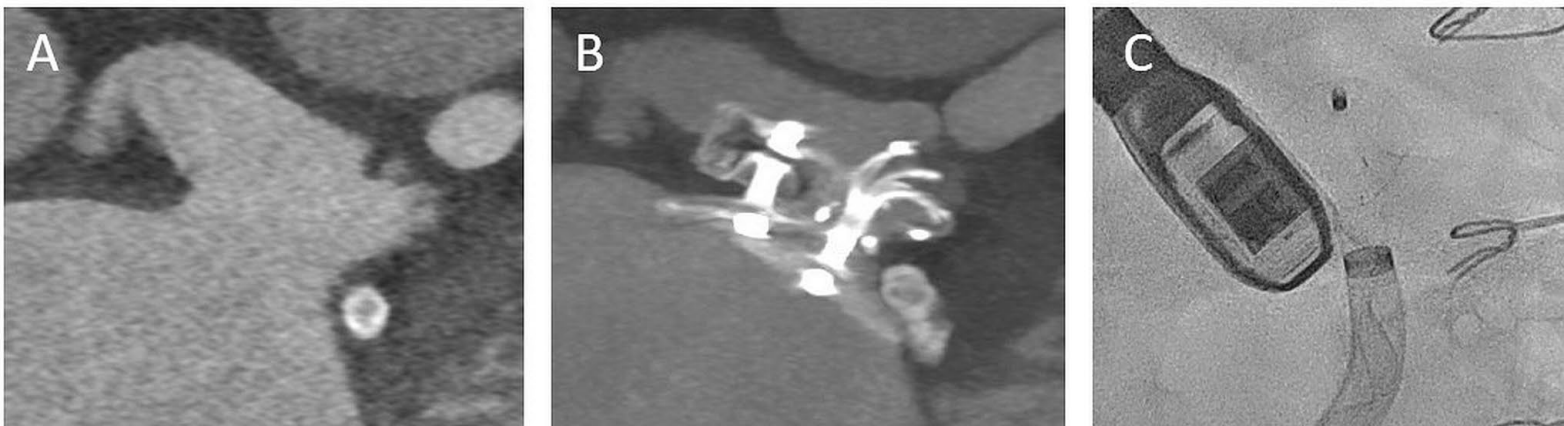
CT-aided planning for complex LAA intervention. An 82-year-old patient admitted for transcatheter left atrial appendage closure due to bleeding events. **A** Complex LAA anatomy with a large atypical orientated lobe in direction of the left atrial roof and a smaller LV orientated side lobe. **B** Intervention using a steerable sheath with 180° inverted implant. **C** After the initial procedure, a small posterior leak was successfully closed with a second occlude

Closure of the left atrial appendage (LAA) represents an alternative to drug-based thromboembolism prophylaxis [45, 46]. Various closure systems are available for interventional and surgical treatment [47]. Echocardiography is the primary imaging modality for LAA assessment, periprocedural guiding, and postprocedural follow-up. In patients with complex anatomy, CT can provide complementary information to transesophageal echocardiography for of morphological assessment, size determination, and device selection (Fig. 4, Table 1) [7•]. In addition, CT allows a comprehensive assessment of adjacent structures, planning of the transseptal puncture site, and prediction of optimal C-arm angulation. Furthermore, a late contrast image can be acquired for ruling out a LAA thrombus. CT data acquisition is typically prospectively triggered in end-diastole. An euvolemic status of the patient with sufficient filling of the LAA must be ensured.

A paravalvular leak can occur after surgical and interventional valve replacement and is associated with increased mortality. CT can be used as adjunctive modality to echocardiography providing detailed information on the location, shape, and size of paravalvular leaks [9].

## Conclusion

CT imaging plays an important role in the work-up of patients presenting with structural heart disease. Novel transcatheter therapies require a comprehensive preprocedural planning including precise device sizing, assessment of calcification and Neo-LVOT for risk determination, virtual device simulation, and prediction of a co-planar fluoroscopic angulation. Furthermore, CT offers complementary information to echocardiography in patients with implanted devices and suspicion for prosthesis thrombosis, degeneration, or endocarditis.

## Abbreviations

CT: Computed tomography
TAVI: Transcatheter aortic valve implantation
LAA: Left atrial appendage
LVOT: Left ventricular outflow tract
MAC: Mitral annular calcification
HALT: Hypoattenuated leaflet thickening
HAM: Hypoattenuation affecting motion
